# Toward learning the rules that predict siRNA efficacy

**DOI:** 10.1016/j.omtn.2023.07.023

**Published:** 2023-08-10

**Authors:** Xavier Bofill-De Ros

**Affiliations:** 1 Department of Molecular Biology and Genetics, Aarhus University, Aarhus, Denmark

In this study, Monopoli et al.^1^ show that machine learning models can be trained to identify robust small interfering RNAs (siRNAs) using small datasets containing fewer than 500 sequences. To overcome the requirement of larger datasets, the authors used an asymmetric trichotomous partitioning. This novel approach allows training machine learning models with experimental data in settings where large datasets are hard to obtain.

Since 2018, three therapeutic siRNAs have been approved for treatment of multiple human diseases, and the seven other candidates reached phase 3 clinical trials. Thus, siRNAs hold great promise as broad RNA-based therapeutics. In order to increase their half-life and availability *in vivo*, therapeutic siRNAs bear extensive chemical modifications. These chemistries can include 2′-ribose modifications (such as 2′-hydroxyl, 2′-fluoro, and 2′-O-methyl), backbone modifications (such as phosphodiester and phosphorothioate), and conjugations with larger moieties. Thus, identifying effective chemically modified siRNA sequences remains a challenge, requiring the assistance of computational algorithms. Different from endogenous small RNAs where extensive knowledge has been gathered regarding the rules that govern duplex unwinding, target recognition, and slicing activity,^2^^,^^3^^,^^4^ not enough is known to forecast the impact of chemical modifications on a given siRNA sequence.

**Figure 1:**
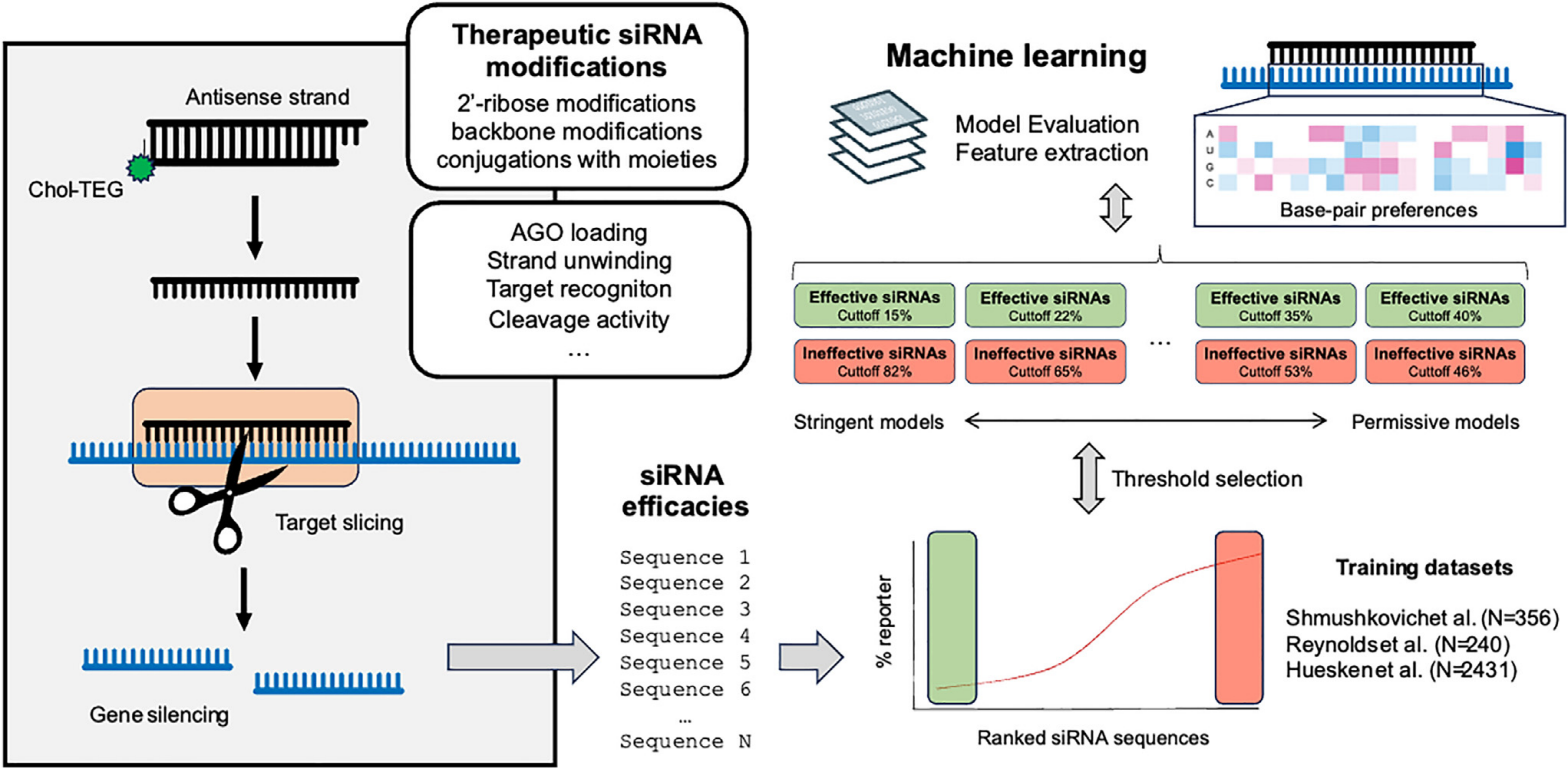
Learning the rules of siRNA efficacy The study by Monopoli al. contributes to our understanding of how machine learning can guide the design and optimization of therapeutic siRNAs. The authors show that tailored machine learning approaches can be used with smaller datasets. The authors also show that random forest models perform better than linear models.

Machine learning has emerged as a powerful tool in biological research; however, its application to predict siRNA efficacy is hindered by the limited size of available datasets. In this study, Monopoli et al. present a framework that applies machine learning to small datasets of modified siRNA sequences for efficacy prediction. To overcome the inherent noise and biological limitations in siRNA datasets, the authors employ a trichotomous partitioning approach. Briefly, this approach consists of generating multiple combinations of siRNA sequences classified into three categories: effective, undefined, and ineffective siRNAs (Figure 1). Their dataset of chemically modified siRNAs includes the repression efficacies of hundreds of sequences targeting 17 different genes. The authors evaluate the effects of different thresholds on the performance of a random forest machine learning model using an evaluation metric that considers class imbalances. For example, the authors evaluate highly stringent models with thresholds set up to learn from the top 15% most effective siRNAs and the bottom 82% least effective siRNAs. Such a stringent model (15/82) did not perform well, due to the reduced size of the dataset. By contrast, more permissive models with threshold pair 22/53 produced better performing models. The authors consider multiple metrics (adjusted AUCPR [precision-recall curve] and contingency table) in order to evaluate final model performances.

Through the analysis, they identify threshold values that yield a predictive model that outperforms linear models trained on the same dataset (using the same classification threshold pairs). In line with previous studies, the random forest model provides higher resolution in capturing these features compared to the linear models. It is reported that random forest models can easily capture complex interactions such as sequence motifs. Similar settings where random forest approaches also performed well include the prediction of base-editing efficiencies^5^ or modeling on-target and off-target CRISPR effects.^6^

In their study, the authors test their learning approach in three independent siRNA datasets from Shmushkovich et al. (N = 356 sequences),^7^ Reynolds et al. (N = 240 sequences),^8^ and Huesken et al. (N = 2,431 sequences).^9^ Furthermore, the authors validate the predictive power of their model through experimental evaluation on a subset of siRNAs predicted to be effective and ineffective. The authors also investigate the features generated by the model using extraction methods. They report the weights of each target position, base, and thermodynamic preferences for an optimal siRNA efficacy. This feature analysis supports the current understanding of the mechanisms underlying siRNA-mediated gene silencing.

The study also points to other important questions for the field. Despite the robustness of models trained in each case, the comparison of the weights of the features extracted from siRNA datasets with or without chemical modifications differs significantly. Based on that, it is also expected that different siRNA chemistries may also result in distinct predictive models. This poses the challenge on how to compare across the different proprietary siRNA compositions used therapeutically or whether leads obtained from unmodified siRNA sequences can be easily turned into therapeutic siRNA. Also, it is intriguing to speculate how different siRNA chemistries could also result in distinct off-target effects or whether machine learning approaches could be used to mitigate undesired off-target effects.

The integration of machine learning with limited siRNA datasets opens new avenues for the development of effective chemically modified siRNAs. By leveraging the power of learning algorithms, the authors exemplify the design and optimization of siRNA-based therapeutics from a large combinatorial space. Overall, this framework is applicable to other classification challenges involving small biological datasets, presenting an opportunity for researchers to develop highly efficient design algorithms.
